# Impact of Epstein–Barr virus infection on clinical outcomes and immune profiles in patients with severe fever with thrombocytopenia syndrome

**DOI:** 10.3389/fimmu.2025.1709364

**Published:** 2026-01-12

**Authors:** Tong Wang, Ling Xu, Hanxin Li, Zhong Zheng, Sumeng Li, Hua Wang, Yanan Liu, Xin Zheng

**Affiliations:** Department of Infectious Diseases, Union Hospital, Tongji Medical College, Huazhong University of Science and Technology, Wuhan, China

**Keywords:** severe fever with thrombocytopenia syndrome, Epstein–Barr virus, co-infection, immune dysregulation, prognosis

## Abstract

**Background:**

Epstein–Barr virus (EBV) infection is frequently observed in patients with severe fever with thrombocytopenia syndrome (SFTS), yet its clinical significance remains unclear. This study aimed to characterize the impact of EBV infection on clinical outcomes and the associated immunological and inflammatory profiles in SFTS patients.

**Methods:**

A retrospective study was conducted on laboratory-confirmed SFTS patients admitted to Wuhan Union Hospital between January 2021 and June 2025, who were categorized according to plasma EBV DNA status at admission. Propensity score matching (PSM) was applied to address baseline imbalances, and Kaplan–Meier analysis was used to evaluate survival differences. Subgroup analyses using logistic regression were performed to assess the effect of EBV co-infection on mortality. Spearman correlation analyses assessed associations between EBV DNA levels and clinical parameters, and ROC analysis evaluated the predictive performance of immune indicators for EBV infection.

**Results:**

Among 306 patients with SFTS, 181 (59.2%) were EBV-positive at admission. EBV-positive patients exhibited significantly higher mortality compared to EBV-negative patients (24.3% vs. 8.0%, P < 0.001), although EBV DNA status was not an independent predictor of mortality after multivariable adjustment (OR = 1.31, 95% CI: 0.54–3.17, P = 0.549). The effect of EBV DNA positivity on mortality was more pronounced in elderly patients, those with delayed admission, or comorbidities. Immunological profiling of EBV-positive patients revealed a marked increase in B-cell frequency alongside pronounced reductions in CD3^+^, CD4^+^, and CD8^+^ T cells, with EBV DNA levels strongly correlating with B-cell proportion. Plasma IL-6 and IL-10 levels were markedly increased in EBV-positive group, with EBV DNA positively correlating with IL-10.

**Conclusion:**

SFTS patients with EBV infection exhibit higher mortality and an immunological profile mirroring that of severe SFTS. Furthermore, EBV DNA may further disrupt B-cell subsets and exacerbate inflammatory dysregulation, thereby intensifying immune dysfunction.

## 1 Introduction

Severe fever with thrombocytopenia syndrome (SFTS) is an emerging infectious disease first identified in China in 2009 and has since been reported in Japan, South Korea, Vietnam, and several other countries (1–3). The causative agent, severe fever with thrombocytopenia syndrome virus (SFTSV), also referred to as Dabie Bandavirus (DBV), belongs to the genus Bandavirus within the family Phenuiviridae (4, 5). Clinically, SFTS is characterized by acute fever, thrombocytopenia and gastrointestinal symptoms. In severe cases, patients may rapidly develop altered consciousness, disseminated intravascular coagulation (DIC), multiple organ failure (MOF), and even death, with mortality rates as high as 30% (4, 6). Despite its severity, there are currently no approved antiviral treatments or vaccines for SFTS. Increasing evidence indicates that dysregulation of the host immune response plays a critical role in SFTS pathogenesis and contributes to adverse clinical outcomes, which may create conditions permissive for viral reactivation (4, 6–9). Epstein–Barr virus (EBV), one of the most ubiquitous human pathogens, establishes lifelong latency by maintaining its genome as circular episomes in host cells, typically without overt clinical manifestations (10–13). However, in the setting of host genetic defects or immunosuppression, EBV can reactivate from its latent state (10). Such reactivation has been documented in association with several clinically relevant viral infections, including dengue virus, SARS-CoV-2, and human immunodeficiency virus (HIV) (11, 14–16). Although EBV co-infection has been observed in SFTS patients, its clinical and immunological implications remain poorly understood. Existing studies are limited by small sample sizes and insufficient analytical depth (11, 13), leaving the impact of EBV co-infection on disease progression unclear. This study systematically investigates the clinical and immunological characteristics of SFTS patients with EBV co-infection and evaluates their prognostic significance.

## 2 Materials and methods

### 2.1 Study design and participants

This retrospective study included patients who were hospitalized with SFTS at the Department of Infectious Diseases, Union Hospital, Wuhan, between January 2021 and June 2025. All enrolled patients underwent testing for both SFTSV RNA and plasma EBV DNA upon admission. A diagnosis of SFTS was confirmed by a positive result for SFTSV RNA using real-time reverse transcription polymerase chain reaction (RT-PCR). Patients were categorized into EBV-positive and EBV-negative groups according to plasma EBV DNA results. Patients meeting any of the following criteria were excluded: age <18 years, coinfection with cytomegalovirus (CMV), human immunodeficiency virus (HIV), hepatitis E virus (HEV), or other acute infectious pathogens; and diagnosis of autoimmune diseases or malignancies. A total of 306 patients met the inclusion criteria and were included in the final analysis, comprising 125 EBV-negative and 181 EBV-positive SFTS cases. The study was approved by the Clinical Ethics Committee of Union Hospital (Approval No. 2025-0888).

### 2.2 Clinical data collection and definition

Clinical data were extracted from electronic medical records using a standardized format by trained physicians. The extracted variables included demographic characteristics, comorbidities, clinical symptoms and signs, laboratory findings, treatments, and outcomes. Missing data for routine laboratory variables were minimal (<5%) and were imputed using the k-nearest neighbors (KNN) method (**Supplementary Figure 1**). Variables with substantial missing data, such as immune cell subsets and cytokines, were not imputed and were analyzed separately in the Results section. Plasma EBV DNA was measured in the hospital’s clinical laboratory using real-time quantitative PCR (qPCR). Patients with a quantifiable plasma EBV DNA load (copies/mL) were classified as EBV-positive, whereas those with undetectable EBV DNA were classified as EBV-negative. A previously developed machine learning–based mortality risk score for SFTS was applied to each patient. This model, established by our team, is available as an online clinical tool (http://175.178.66.58/english/) (17).

### 2.3 Statistical analysis

Continuous variables were presented as median (interquartile range, IQR) or mean ± standard deviation (SD) and compared using the Mann–Whitney U test or Student’s t test. Categorical variables were expressed as frequencies and proportions and analyzed using the chi-square or Fisher’s exact test. Spearman’s rank correlation coefficient was used to assess correlations between continuous variables. To adjust for confounding and reduce selection bias, propensity score matching (PSM) was performed. Propensity scores were estimated using a logistic regression model including four baseline covariates, and 1:1 nearest-neighbor matching was applied with a caliper of 0.2 to balance baseline characteristics between groups. Kaplan–Meier survival curves were generated and compared between groups using the log-rank test. Univariate and multivariate logistic regression analyses were performed to evaluate the association between EBV infection and mortality among SFTS patients. Subgroup analyses were conducted using stratified logistic regression models to assess potential heterogeneity, and interactions were examined with likelihood ratio tests. Statistical significance was defined as a two-sided p < 0.05, with increasing significance denoted as p < 0.05, p < 0.01, and p < 0.001. All analyses were performed using SPSS (version 27.0), R (version 4.4.1), and GraphPad Prism (version 9.5.0).

## 3 Results

### 3.1 Baseline characteristics of SFTS patients with and without EBV infection

A total of 306 patients with laboratory-confirmed severe fever with thrombocytopenia syndrome (SFTS) upon admission and available plasma Epstein-Barr virus (EBV) DNA test results were enrolled in this study. The median age was 65 years (interquartile range [IQR]: 58–70 years), with 56.9% being female. Comorbidities were present in 37.9% of the cohort, most commonly hypertension, coronary artery disease, cerebrovascular disease, and diabetes. Patients were classified based on plasma EBV DNA status, with 181 (59.2%) being EBV DNA–positive and 125 (40.8%) being EBV DNA–negative. The overall mortality rate was 17.6% (54/306). Baseline demographic and laboratory characteristics are presented in **Table 1**. Compared with EBV DNA-negative patients, those who were positive were significantly older (66 vs. 62 years, p = 0.012) and had a longer interval from symptom onset to hospitalization (7.0 vs. 4.5 days, p < 0.001). The prevalence of hypertension, coronary heart disease, and cerebrovascular disease did not differ significantly between groups (all p > 0.05), whereas diabetes was more common in the EBV DNA-positive group. Neurological manifestations were more frequent in EBV DNA–positive patients (45.9% vs. 34.4%, p = 0.045), while respiratory and gastrointestinal symptoms were similarly distributed between the groups. Laboratory analyses revealed that EBV DNA-positive patients had significantly higher levels of SFTSV viral load, ferritin, alanine aminotransferase (ALT), aspartate aminotransferase (AST), creatine kinase (CK), lactate dehydrogenase (LDH), blood urea nitrogen (BUN), creatinine, D-dimer, activated partial thromboplastin time (APTT), and thrombin time (TT) (all p < 0.05). In contrast, platelet count and serum albumin level were significantly lower in the EBV DNA-positive group (all p < 0.05). No significant differences were observed in total white blood cell count or the absolute and relative proportions of lymphocytes, monocytes, and neutrophils between the two groups (**Table 1**).

**Table 1:** Clinical and laboratory characteristics of patients with severe fever with thrombocytopenia syndrome with and without EBV co-infection.

Variable	Total (n=306)	SFTS/EBV^-^ (n=125)	SFTS/EBV^+^ (n=181)	P-value
Demographic data				
Age (years)	65 (58–70)	62 (57–69)	66 (59–71)	0.012
Gender female	56.9 (174/306)	56.0 (70/125)	57.5 (104/181)	0.80
Time from onset to admission, days (IQR)	7 (5–7)	4.5 (5–7)	7 (5–7)	<0.001
Death	17.6 (54/306)	8.0 (10/125)	24.3 (44/181)	<0.001
Pre-existing comorbidity	37.9 (116/306)	35.2 (44/125)	39.8 (72/181)	0.417
DM	13.1 (40/306)	8.0 (10/125)	16.6 (30/181)	0.029
Hypertension	25.8 (79/306)	25.6 (32/125)	26.0 (47/181)	0.943
Cardiovascular disease	4.9 (15/306)	4.0 (5/125)	5.5 (10/181)	0.544
Cerebrovascular disease	4.2 (13/306)	4.8 (6/125)	3.9 (7/181)	0.691
Symptoms before or at the beginning of treatment, n (%)				
Gastrointestinal symptoms	69.0 (211/306)	69.6 (87/125)	68.5 (124/181)	0.839
Respiratory tract symptoms	35.6 (109/306)	29.6 (37/125)	39.8 (72/181)	0.068
Nervous systems	41.2 (126/306)	34.4 (43/125)	45.9 (83/181)	0.045
Laboratory parameters				
Mortality risk (%)	2.13 (0.64-10.31)	0.85 (0.31-2.81)	4.02 (1.18-20.79)	<0.001
Virus load (Log10)	3.20 ± 1.42	2.55 ± 1.18	3.64 ± 1.39	<0.001
Ferritin (ug/L)	9435.0 (4053.0-24776.0)	4718.5 (2491.9-11867.4)	12732.3 (6687.9-39352.3)	<0.001
WBC (10^9/L)	3.22 (1.95-5.60)	3.12 (1.87-5.52)	3.30 (2.10-5.60)	0.449
PLT (10^9/L)	48 (33–65)	54 (37–73)	43 (32–60)	<0.001
HB (g/L)	127 ± 17.31	127 ± 15.77	127 ± 18.35	0.994
NEUT (10^9/L)	2.13 (1.07-4.05)	2.05 (1.0-3.95)	2.17 (1.10-4.08)	0.394
LYM (10^9/L)	0.68 (0.45-1.16)	0.68 (0.40-1.11)	0.68 (0.48-1.17)	0.499
MONO (10^9/L)	0.15 (0.07-0.32)	0.16 (0.08-0.34)	0.14 (0.07-0.31)	0.479
NEUT (%)	70.0 (51.9-82.1)	75.2 (46.3-84.0)	69.0 (54.0-80.0)	0.824
LYM (%)	22.0 (14.0-36.4)	19.7 (12.9-41.1)	23.3 (14.8-34.6)	0.922
MONO (%)	4.45 (2.50-8.83)	4.8 (3.0-9.8)	4.2 (2.3-8.6)	0.274
ALT, U/L	77 (47–133)	67 (40–112)	88.0 (54.0-152.5)	<0.001
AST (U/L)	191 (108–346)	134 (80–223)	244 (136–474)	<0.001
ALB (g/L)	31.61 ± 4.04	32.65 ± 4.18	30.90 ± 3.79	<0.001
CK	481.5 (227.8-1057.3)	372.0 (185.0-948.5)	565.0 (280.0-1084.0)	0.005
LDH (U/L)	740.0 (492.0-1229.8)	517.0 (391.5-780.0)	902.0 (622.5-1403.0)	<0.001
BUN (mmol/lL)	5.09 (3.71-6.55)	4.60 (3.49-6.18)	5.33 (3.95-7.04)	0.002
CREA (umol/L)	71.0 (59.0-84.0)	68.1 (57.9-80.2)	72.9 (60.1-90.7)	0.022
D-dimer (mg/L)	2.70 (1.55-6.56)	1.90 (1.21-3.98)	3.60 (1.20-8.28)	<0.001
APTT (s)	49.8 (44.0-60.8)	47.4 (41.7-53.3)	54.5 (46.1-65.0)	<0.001
TT(s)	26.0 (22.3-32.6)	23.2 (20.8-26.8)	29.0 (24.3-40.9)	<0.001

Group labels: SFTS/EBV+ refers to SFTS patients with detectable EBV DNA in plasma; SFTS/EBV– refers to those without.

SFTS, severe fever with thrombocytopenia syndrome; WBC, white blood cells; HB, hemoglobin; PLT, platelets; NEUT, neutrophils; LYM, lymphocytes; MONO, Monocytes; ALT, alanine Aminotransferase; AST, aspartate Aminotransferase; ALB, albumin; CK, creatine kinase; LDH, lactate dehydrogenase; BUN, blood urea nitrogen; CREA, creatinine; APTT, activated partial thromboplastin time; TT, thrombin time.

**Figure 1:**
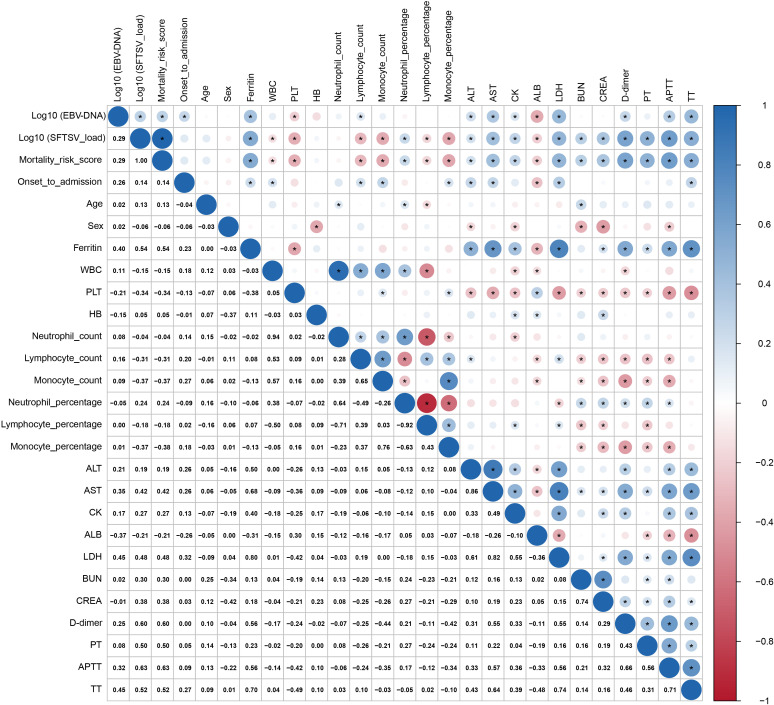
Correlation analyses between EBV DNA levels and laboratory parameters in SFTS patients. An asterisk (*) denotes correlations with P < 0.05.

To further evaluate the clinical relevance of plasma EBV DNA levels, we assessed their associations with key laboratory parameters. The analysis revealed that EBV DNA levels were significantly associated with higher SFTSV viral load, mortality risk score, time from symptom onset to hospitalization, ferritin, lymphocyte count, ALT, AST, CK, LDH, D-dimer, APTT, and TT (ferritin: r = 0.40; AST: r = 0.35; LDH: r = 0.45; APTT: r = 0.32; TT: r = 0.45; all p < 0.05), and negatively correlated with PLT (r = -0.21) and ALB (r = -0.37) (both p < 0.05)(**Figure 1**).

### 3.2 Impact of EBV infection on clinical outcome in SFTS patients

**Figure 2:**
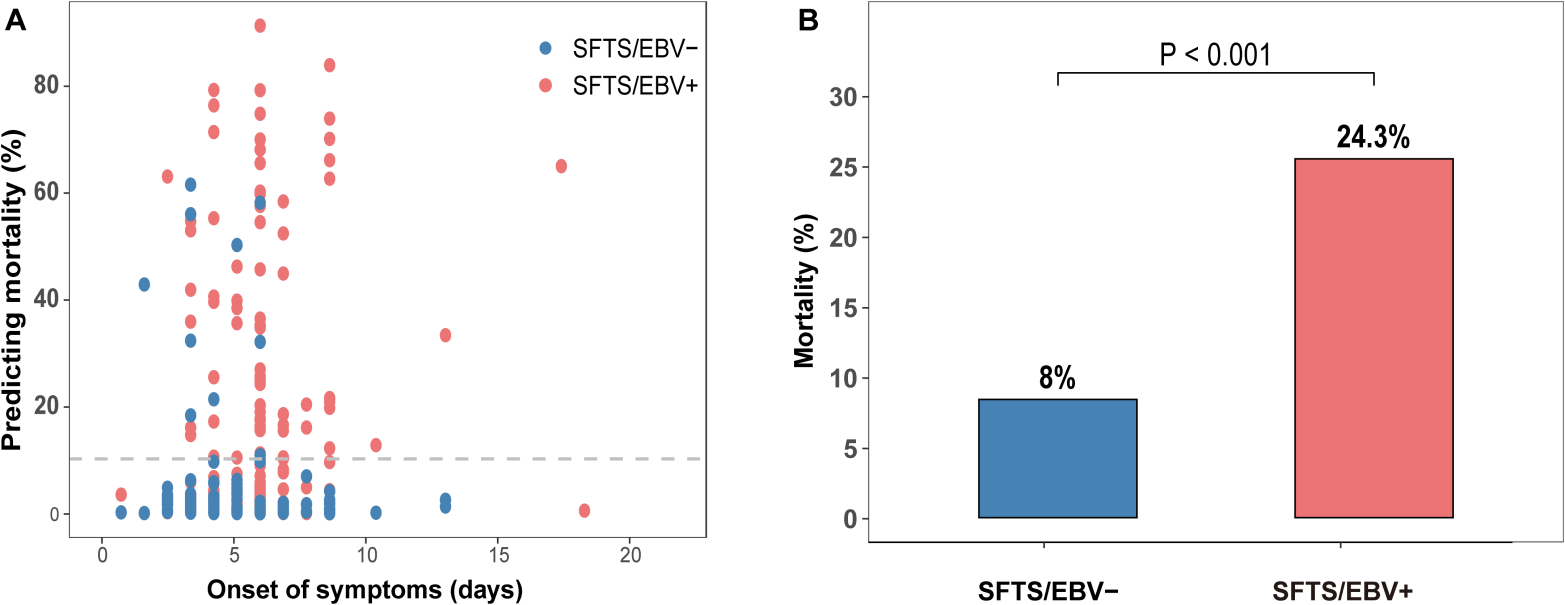
Comparison of predicted mortality risk and observed mortality between EBV-positive and EBV-negative groups among patients with SFTS. SFTS/EBV+ refers to SFTS patients with detectable EBV DNA in plasma; SFTS/EBV– refers to those without.

The above results showed that SFTS patients with EBV infection exhibited more severe multi-organ dysfunction, with elevated biomarkers observed in systems such as the liver, kidneys, and coagulation cascade. To further evaluate the clinical implications of EBV reactivation in SFTS, we applied our previously established severity prediction model and found a significantly higher mortality risk in patients with EBV infection compared to those without (4.02% vs. 0.85%, P < 0.001) (**Figure 2**). Consistently, the all-cause mortality rate was significantly higher in the EBV-positive group (24.3% vs. 8.0%, P < 0.001). In the unmatched cohort, Kaplan-Meier survival analysis revealed a significantly lower 30-day survival probability among SFTSV/EBV positive patients relative to EBV-negative counterparts (76.8% vs. 92.0%, log-rank P<0.001) (**Supplementary Figure 3A**). To account for potential confounding due to baseline imbalances, propensity score matching (PSM) was conducted using key covariates, including baseline mortality risk score, sex, time from symptom onset to hospitalization, and comorbidities (**Supplementary Figure 2**). After matching, no significant difference in 30-day survival was observed between the two groups (91.13% vs. 91.90%, log-rank P=0.869) (**Supplementary Figure 3B**). In addition, a multivariable logistic regression model incorporating EBV status, platelet count, serum albumin level, and mortality risk score as covariates revealed that only the mortality risk score remained independently associated with mortality, whereas EBV status showed no significant association (OR=1.31, 95% CI: 0.54-3.17, P=0.549) (**Supplementary Table 1**), suggesting that EBV reactivation does not independently contribute to mortality in patients with SFTS.

### 3.3 Effect of EBV infection on mortality in different subgroups of patients with SFTS

**Figure 3:**
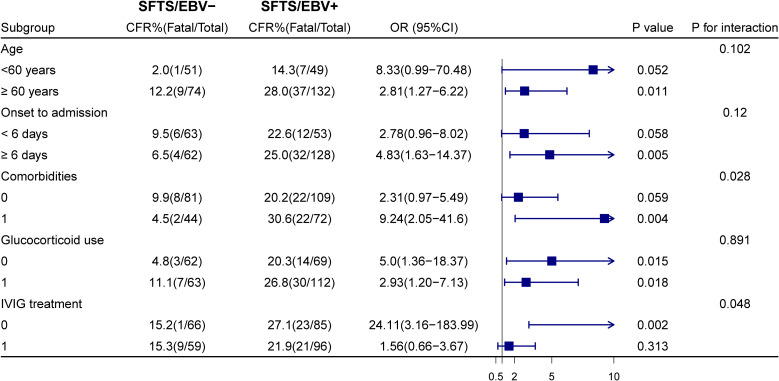
Subgroup analyses of the association between EBV co-infection and mortality in patients with SFTS.

To further evaluate the impact of EBV reactivation on mortality various clinical subgroups of SFTS, we conducted stratified analyses according to age, time from symptom onset to hospitalization, presence of underlying comorbidities, glucocorticoid therapy, and intravenous immunoglobulin (IVIG) treatment (**Figure 3**). EBV-DNA positivity was significantly associated with an increased risk of mortality in several subgroups, including patients aged ≥60 years (HR = 2.36, 95% CI: 1.14–4.91, P = 0.021), those hospitalized ≥6 days after symptom onset (HR = 5.98, 95% CI: 1.42–25.16, P = 0.015), individuals with preexisting comorbidities (HR = 7.28, 95% CI: 1.71–31.0, P = 0.007), and those not receiving IVIG during hospitalization. Furthermore, the elevated mortality risk associated with EBV-DNA positivity remained significant regardless of glucocorticoid use.

Interaction analyses further revealed significant interactions between EBV-DNA positivity and both comorbidity status (P for interaction = 0.028) and IVIG treatment (P for interaction = 0.048), whereas no significant interactions were observed for the remaining stratification variables (**Figure 3**).

### 3.4 EBV-associated immune cell and cytokine alterations in SFTS patients

Given the potential association between EBV infection and host immune dysregulation, we performed an exploratory analysis of 122 patients in the overall cohort who had available lymphocyte subset data. As shown in **Supplementary Table 2**, the distribution of disease severity in this subgroup was comparable to that of the full cohort. **Supplementary Table 3** further demonstrates that their baseline profiles aligned with the overall pattern: patients in the EBV-positive group were older, had a longer symptom-onset–to-admission interval, and more frequently exhibited neurological manifestations. In addition, they had substantially higher mortality risk scores, SFTSV viral load, and mortality compared with EBV-negative patients.

**Figure 4:**
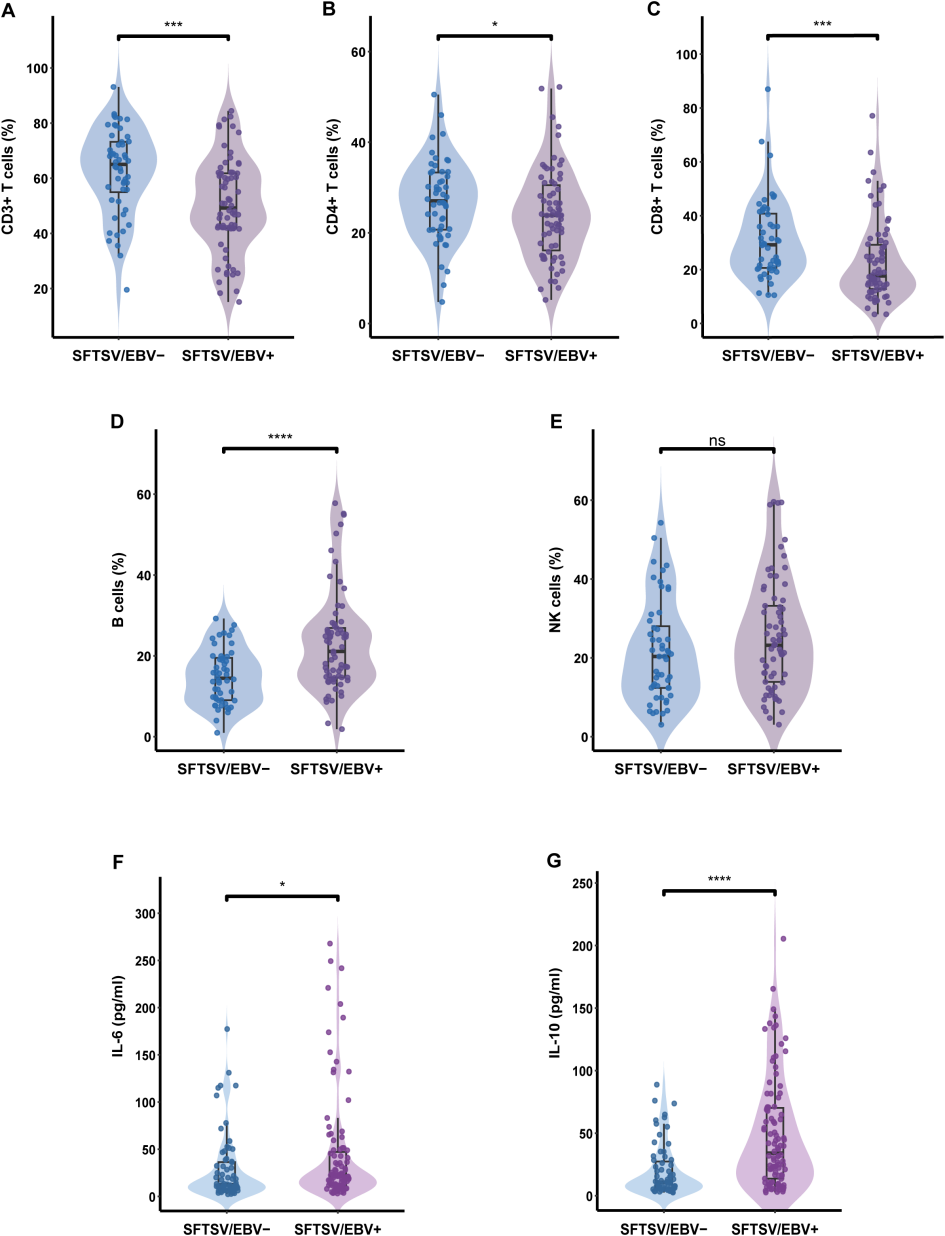
Comparison of immune cell percentages and cytokine levels between EBV-positive and EBV-negative SFTS patients. **(A)** CD3+T cells (%), **(B)** CD4+T cells (%), **(C)** CD8+T cells (%), **(D)** B cells (%), **(E)** NK cells (%), **(F)** IL-6 (pg/ml), **(G)** IL-10 (pg/ml). Group labels: SFTS/EBV+ refers to SFTS patients with detectable EBV DNA in plasma; SFTS/EBV– refers to those without. ns, not significant, *P < 0.05, **P < 0.01, ***P < 0.001, ****P < 0.0001.

**Figure 5:**
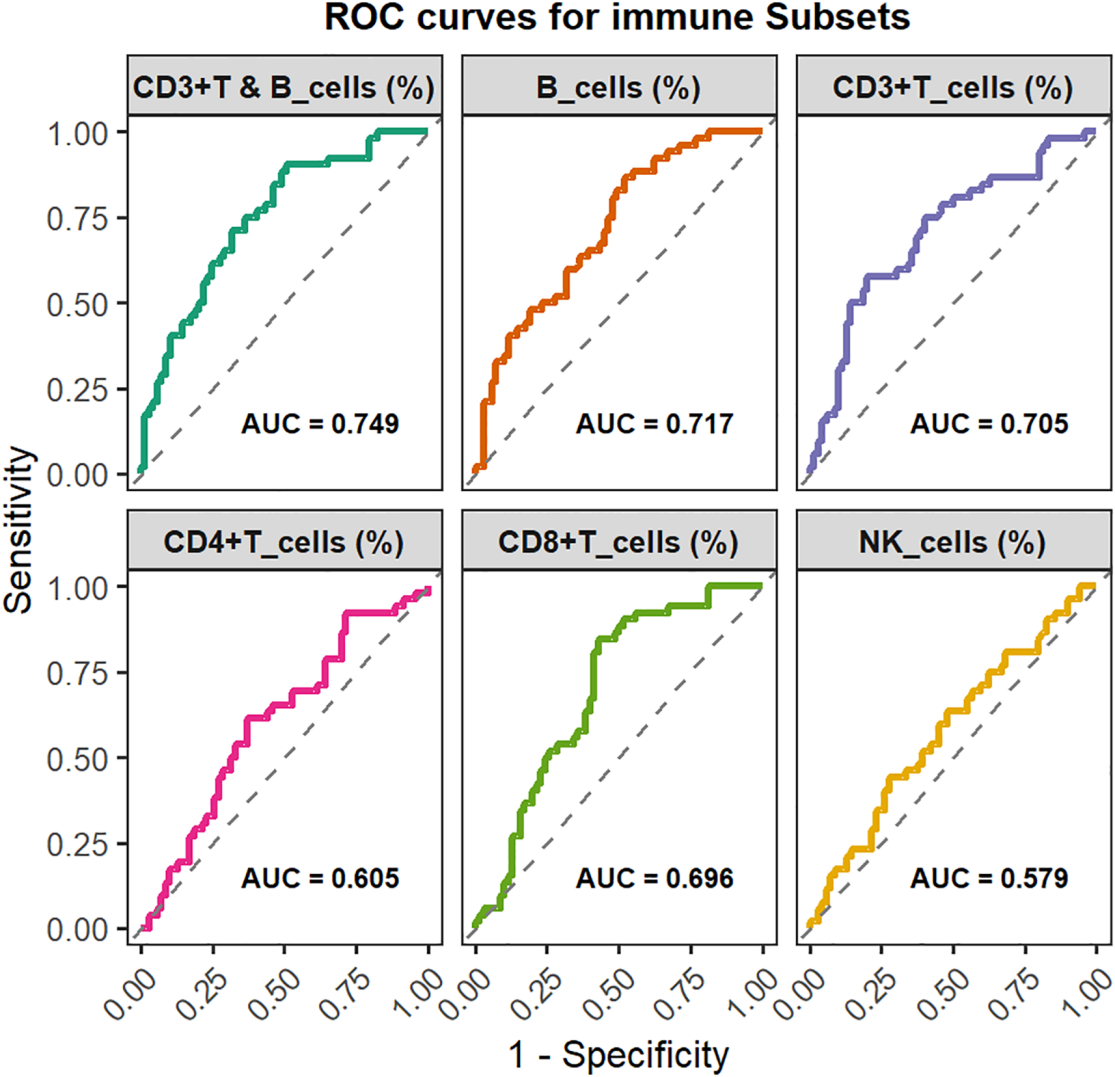
Predictive value of immune cell proportions for EBV infection: ROC curve analysis.

Analysis of lymphocyte subset revealed that the EBV-positive group exhibited a marked reduction in the proportions of CD3+, CD4+ and CD8+ T cells, accompanied by a pronounced increase in B-cell proportions (all P < 0.05). NK-cell proportions were slightly elevated in EBV-positive patients but did not reach statistical significance (**Figures 4A-E**). With respect to absolute counts, EBV-positive patients displayed substantially lower CD3+ and CD8+ T-cell counts, while B cell counts were notably increased. No significant differences were observed in the absolute counts of CD4+ T cells or NK cells between the two groups (**Supplementary Figure 4**). To evaluate the discriminatory capacity of lymphocyte subsets for identifying EBV co-infection, ROC analyses were conducted. B-cell proportion exhibited the highest discriminative performance (AUC = 0.717), followed by CD3+ T-cell proportion, and their combination further improved predictive accuracy (AUC = 0.749) (**Figure 5**).

For cytokine profiling, 168 patients with available measurements were included. EBV-positive patients exhibited significantly higher levels of IL-6 and IL-10 (both P < 0.05) (**Figures 4F-G**), whereas IL-2, IL-4, IFN-γ, and TNF-α concentrations did not differ significantly between groups (**Supplementary Figure 5**). Correlation analyses further revealed a robust positive association between EBV DNA load and B-cell proportion (r = 0.42, P < 0.001), with no significant correlations found for CD3+, CD4+, CD8+, or NK-cell proportions. Furthermore, EBV DNA load was positively correlated with IL-10 and inversely correlated with IL-2 concentrations (**Supplementary Table 4**).

## 4 Discussion

Immune dysregulation and cytokine storm induced by SFTSV infection are central drivers of disease severity (4, 18–20). This dysregulated immune state impairs antiviral surveillance, promoting reactivation of latent viruses such as EBV. The clinical significance of EBV reactivation during SFTS remains poorly characterized. Leveraging the largest reported SFTS–EBV cohort to date, this study provides a systematic evaluation of the clinical relevance of EBV reactivation.

Using the prognostic model for SFTS previously developed by our team (17), we found that patients with EBV co-infection exhibited higher mortality risk scores and a greater observed mortality rate compared with EBV-negative patients, consistent with prior reports (11, 13). Nevertheless, after adjusting for key baseline confounders, EBV-DNA positivity was not independently associated with death, suggesting that it primarily reflects the underlying severity of illness rather than directly contributing to adverse outcomes. To further elucidate its clinical relevance, we examined the associations between EBV DNA load and key laboratory parameters. EBV DNA levels increased in parallel with markers of systemic inflammation and multi-organ injury, showing positive correlations with ferritin, AST, LDH, and APTT, and negative correlations with platelet count and serum albumin (17, 21–23). These coordinated laboratory changes further highlight the strong association between EBV reactivation and severe disease in SFTS.

Subgroup analysis revealed that EBV-positive individuals had significantly higher mortality risk among elderly patients, those with underlying comorbidities, or patients with delayed hospital admission. Given that these clinical characteristics are established indicators of poor prognosis (4, 22), the results suggest that the detrimental effects of EBV infection are particularly pronounced in SFTS patients with a weakened immune baseline or delayed treatment. Furthermore, the association between EBV-DNA positivity and increased mortality was stronger among patients who did not receive IVIG therapy, indicating a potential modulatory role of immunoglobulins in mitigating EBV-related adverse outcomes. Immunoglobulin contains virus-specific antibodies that can promote viral clearance and suppress pro-inflammatory mediators, supporting its dual antiviral and immunomodulatory role (24). Although previous study suggested that immunosuppressive therapy may trigger EBV reactivation (25), EBV-positive patients in our cohort had worse outcomes regardless of corticosteroid use, indicating that the detrimental effects of EBV are more likely driven by host immune status and viral reactivation itself rather than corticosteroid therapy.

SFTSV infection causes severe impairment of cellular and humoral immunity (8, 9, 18, 26), whereas EBV clearance depends on intact innate and adaptive immune responses. In our cohort, EBV-positive patients exhibited significantly lower levels of CD3+, CD4+, and CD8+ T lymphocytes. Since CD8+ T cells are essential for controlling EBV replication, their depletion likely undermines antiviral immune control (10). Concurrently, EBV-positive patients exhibited a marked elevation of B lymphocytes, with EBV DNA levels strongly correlating with B cell frequency. Given that B lymphocytes serve as a shared target for both SFTSV and EBV (9, 10, 27), disrupted B cell homeostasis may play a central role in the pathogenic interplay between the two viruses. Although NK cell levels did not differ significantly between groups, a modest increase was observed in EBV-positive patients, potentially reflecting reactive NK cell proliferation in severe SFTS (13, 28). Overall, the observed immune alterations are primarily associated with the severity of SFTS, and EBV reactivation may act as an amplifying factor that exacerbates pre-existing immune dysregulation, particularly within the B cell compartment.

Analysis of inflammatory cytokines revealed elevated levels of IL-6 and IL-10 in EBV-positive patients, consistent with their more severe clinical presentation (4, 29). Notably, EBV DNA levels were positively associated with IL-10, likely mediated by EBV-encoded latent membrane protein 1 (LMP1), whose expression in infected B cells is a key driver of IL-10 induction (30), indicating that EBV may directly regulate the inflammatory–immune balance and contribute to immunosuppression. Furthermore, EBV DNA levels were negatively correlated with IL-2, a cytokine predominantly produced by CD4+ T cells (31), which were significantly reduced in EBV-positive patients. Despite the lack of a direct correlation between EBV DNA and CD4+ T cell counts, the reduction in IL-2 may reflect an indirect suppressive effect of EBV on CD4+ T cell function, thereby exacerbating immune dysregulation. Overall, our findings suggest a potential reciprocal pathological interplay between SFTSV and EBV in the context of immune dysregulation: SFTSV-induced immunosuppression may facilitate EBV reactivation, while EBV reactivation could further exacerbate immune dysfunction, collectively driving disease progression.

This study has several limitations. As a single-center retrospective analysis, our investigation was primarily based on baseline data collected at hospital admission. Incorporating serial measurements of SFTSV load, EBV DNA levels, and immune and cytokine profiles would have further strengthened the study, which was practically challenging due to the limited financial resources of the predominantly rural patient population. Despite these limitations, our findings provide valuable insights into the clinical and immunological characteristics of SFTS and highlight important directions for future research.

In summary, our study provides the first systematic characterization of the clinical and immunological features of patients with SFTS complicated by EBV infection. Patients with EBV-DNA positivity exhibited higher mortality, although it is more likely a marker of disease severity rather than a direct causal factor. The immunological profile resembles that of severe SFTS, characterized by profound T cell depletion and increased B cell proportion and absolute count. The expanded B cell population may serve as a reservoir for both viruses and could facilitate early detection of EBV reactivation. EBV co-infection may further exacerbate inflammatory dysregulation, highlighting the risk of cytokine storm and humoral immune perturbation. Immunoglobulin therapy may provide potential clinical benefit in this patient population. These findings advance our understanding of the immunopathological mechanisms underlying SFTS–EBV co-infection and inform early risk stratification and personalized therapeutic strategies. In the future, prospective studies are warranted to determine whether EBV DNA levels provide prognostic value independent of established severity indicators and to define the optimal indications and timing of immunoglobulin therapy in patients with concurrent SFTSV and EBV infection.

## Data Availability

The original contributions presented in the study are included in the article/**Supplementary Material**. Further inquiries can be directed to the corresponding author/s.
