# Supplementary material for: Impact of Epstein–Barr virus infection on clinical outcomes and immune profiles in patients with severe fever with thrombocytopenia syndrome
Source: Front Immunol. 2026 Jan 12;16:1709364. doi: 10.3389/fimmu.2025.1709364 (PMC12833342; doi:10.3389/fimmu.2025.1709364)
Supplement: Supplementary file 1 [file Supplementaryfile1.docx]

**Impact of Epstein–Barr virus infection on clinical outcomes and immune profiles in patients with severe fever with thrombocytopenia syndrome**

Tong Wang MD^1, *^, Ling Xu MD^1, *^, Hanxin Li MD^1^, Zhong Zheng MD^1^, Sumeng Li MD^1^, Hua Wang BD ^1^, Yanan Liu MD^1^, Xin Zheng PhD ^1, #^

^1^ Department of Infectious Diseases, Union Hospital, Tongji Medical College, Huazhong University of Science and Technology, Wuhan 430022, China.

* Tong Wang and Ling Xu contributed equally to this study.

^#^Corresponding Authors.

Prof. Xin Zheng

E-mail: [xinz@hust.edu.cn](mailto:xinz@hust.edu.cn)

Tel: 86-(027)-85726978

Department of Infectious Diseases, Union Hospital, Tongji Medical College, Huazhong University of Science and Technology, Wuhan 430022, China

**Supplementary Digital Material**

**Table of Contents**

[Table S1. Multivariate logistic regression analysis on the risk factors associated with mortality of SFTS. 2](#_Toc166962132)

[Table S2: Comparison of baseline characteristics between the overall cohort and the subset of patients with available immune cell data. 3](#_Toc166962135)

[Table S3: Baseline characteristics and outcomes of SFTS patients with immune cell subset data, compared by EBV status 3](#_Toc166962136)

[Table S4: Spearman’s correlation of EBV DNA (log10 copies/mL) with immune cell percentages and cytokine levels in SFTS patients. 4](#_Toc166962137)

[Supplementary Figure 1: Overview of missing data patterns among variables in the SFTS patient dataset. 5](#_Toc166962138)

[Supplementary Figure 2: Standardized mean differences of baseline characteristics between EBV-positive and EBV-negative groups before and after propensity score matching (PSM) adjustment. 6](#_Toc166962139)

[Supplementary Figure 3: Kaplan–Meier survival curves of SFTS patients with and without EBV co-infection before and after adjustment using PSM. 6](#_Toc166962139)

[Supplementary Figure 4: Comparison of immune cell counts and cytokine levels between EBV-positive and EBV-negative SFTS patients.](#_Toc166962139) 7

[Supplementary Figure 5: Comparison of cytokine levels between EBV-positive and EBV-negative SFTS patients.](#_Toc166962139) 8

**Table S1. Multivariate logistic regression analysis on the risk factors associated with mortality of SFTS.**

| Variants | Univariate analysis | | Multivariate analysis | | |  |  |
| --- | --- | --- | --- | --- | --- | --- | --- |
|  | OR (95%CI) | *P-value* | OR (95%CI) | *P-value* | |  |  |
| SFTS/EBV+ | 3.69 (1.78-7.66) | ＜0.001 | 1.31 (0.54-3.17) | | 0.549 | |  |
| Mortality risk score (%) | 1.07 (1.06-1.09) | ＜0.001 | 1.07 (1.05-1.09) | | ＜0.001 | |  |
| Sex | 1.13 (0.62-2.05) | 0.695 |  | |  | | |
| Days from onset to admission | 1.006 (0.94-1.08) | 0.873 |  | |  | | |
| Comorbidities | 1.39 (0.77-2.52) | 0.277 |  | |  | | |
| PLT (109/L) | 0.96 (0.95-0.98) | ＜0.001 | 0.98 (0.96-1.004) | | 0.116 | | |
| NEUT (10^9/L) | 0.97 (0.87-1.09) | 0.637 |  | |  | | |
| LYM (10^9/L) | 0.88 (0.56-1.40) | 0.595 |  | |  | | |
| MONO (109/L) | 0.73 (0.24-2.23) | 0.577 |  | |  | | |
| ALB (g/L) | 0.84 (0.77-0.91) | ＜0.001 | 0.91 (0.83-1.01) | | 0.071 | | |
| Antiviral treatment | 1.67 (0.77-3.60) | 0.194 |  | |  | | |
| IVIG treatment | 1.27 (0.70-2.29) | 0.428 |  | |  | | |
| Glucocorticoid use | 1.80 (0.96-3.36) | 0.066 |  | |  | | |

Abbreviations: SFTS, severe fever with thrombocytopenia syndrome; SFTS/EBV+, SFTS patients with detectable EBV DNA in plasma; PLT, platelets; NEUT, neutrophils; LYM, lymphocytes; MONO, monocytes; ALB, albumin; IVIG, intravenous immunoglobulin.

**Table S2. Comparison of baseline characteristics between the overall cohort and the subset of patients with available immune cell data.**

| Variables | Overall cohort  (n=306) | Immune subset  (n=122) | P value |
| --- | --- | --- | --- |
| Age | 65 (58-70) | 66 (58-71) | 0.231 |
| Sex (Female) | 56.9 (174/306) | 50.8 (62/122) | 0.256 |
| Comorbidities | 37.9 (116/306) | 43.4 (53/122) | 0.290 |
| Onset to admission | 7 (5-7) | 7 (5-7) | 0.874 |
| Mortality risk score (%) | 2.13 (0.64-10.31) | 2.30 (0.60-8.96) | 0.876 |
| Virus load (Log10) | 3.20±1.42 | 3.17±1.40 | 0.844 |
| Death | 54 (17.6) | 16 (13.1) | 0.252 |

**Table S3. Baseline characteristics and outcomes of SFTS patients with immune cell subset data, compared by EBV status.**

| Variables | Total (n=122) | SFTS/EBV^-^ (n=52) | SFTS/EBV^+^  (n=70) | P- Value |
| --- | --- | --- | --- | --- |
| Age (years) | 66 (58-71) | 61 (56-70) | 69 (63-72) | 0.002 |
| Gender female | 50.8 (62/122) | 46.2 (24/52) | 54.3 (38/70) | 0.374 |
| Time from onset to admission, days (IQR) | 7 (5-7) | 6 (5-7) | 7 (5-8) | 0.017 |
| Pre-existing comorbidity | 37.9 (116/306) | 35.2 (44/125) | 39.8 (72/181) | 0.880 |
| Nervous systems | 48.4 (59/122) | 34.6 (18/52) | 58.6 (41/70) | 0.009 |
| Mortality risk score (%) | 2.3 (0.60-9.0) | 0.77 (0.30-2.43) | 4.47 (1.85-18.76) | ＜0.001 |
| Virus load (Log10) | 3.18 (2.32-4.12) | 2.65 (1.71-3.27) | 3.76 (2.84-4.64) | ＜0.001 |
| Death | 13.1 (16/122) | 3.8 (2/52) | 20.0 (14/70) | 0.009 |

Abbreviations: SFTS, severe fever with thrombocytopenia syndrome; EBV, Epstein–Barr virus.

**Table S4. Spearman’s correlation of EBV DNA (log10 copies/mL) with immune cell percentages and cytokine levels in SFTS patients.**

| Parameter | n | r | P value |
| --- | --- | --- | --- |
| CD3+T cells (%) | 70 | -0.219 | 0.068 |
| CD4+T cells (%) | 70 | -0.122 | 0.316 |
| CD8+T cells (%) | 70 | -0.15 | 0.214 |
| **B cells (%)** | **69** | **0.415** | **＜0.001** |
| NK cells (%) | 69 | -0.121 | 0.324 |
| IL-6 | 99 | 0.197 | 0.051 |
| **IL-10** | **100** | **0.233** | **0.019** |
| IL-2 | 100 | -0.227 | 0.023 |
| IL-4 | 100 | -0.142 | 0.16 |
| TNF-α | 100 | 0.188 | 0.061 |
| IFN-γ | 100 | 0.048 | 0.633 |

Abbreviations: SFTS, severe fever with thrombocytopenia syndrome; EBV, Epstein–Barr virus.

**Supplementary Figure 1. Overview of missing data patterns among variables in the SFTS patient dataset.**


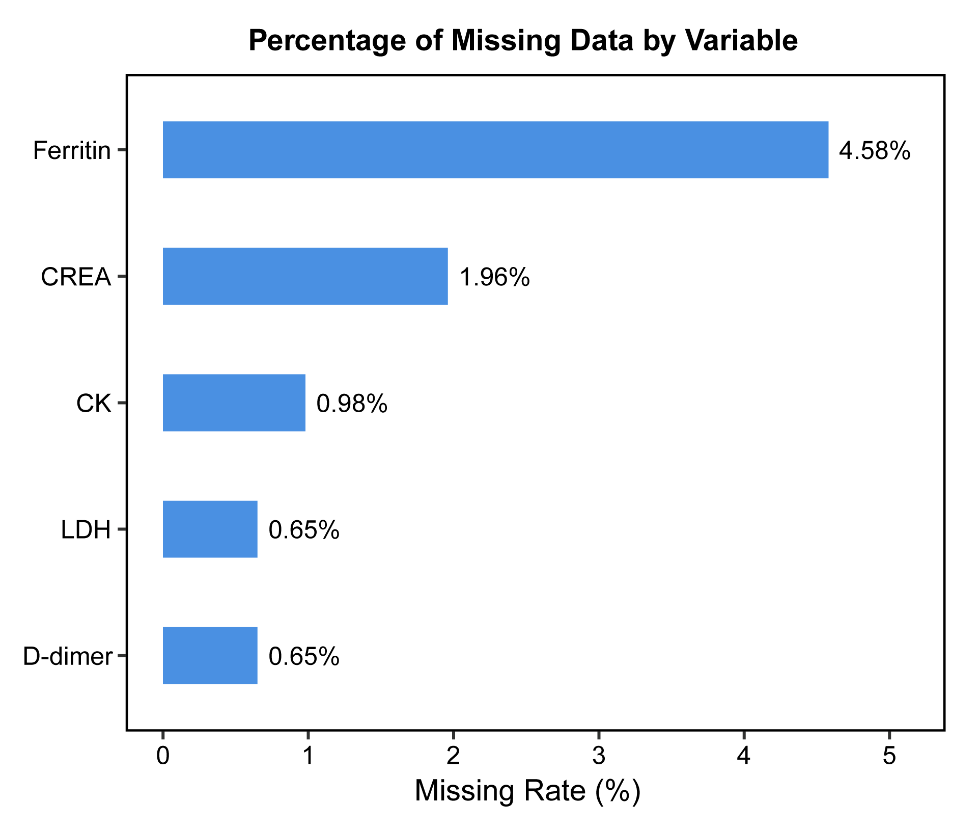


**Supplementary Figure 2. Standardized mean differences of baseline characteristics between EBV-positive and EBV-negative groups before and after propensity score matching (PSM) adjustment.**

**
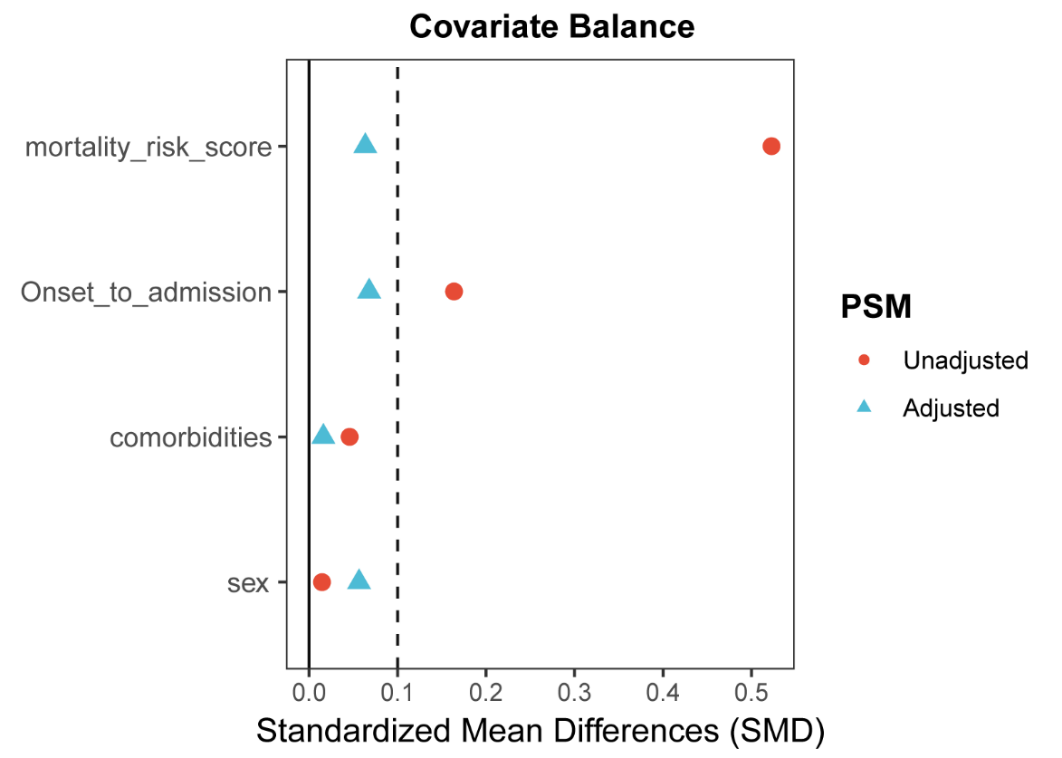
**

**Supplementary Figure 3. Kaplan–Meier survival curves of SFTS patients with and without EBV co-infection before and after adjustment using PSM.**


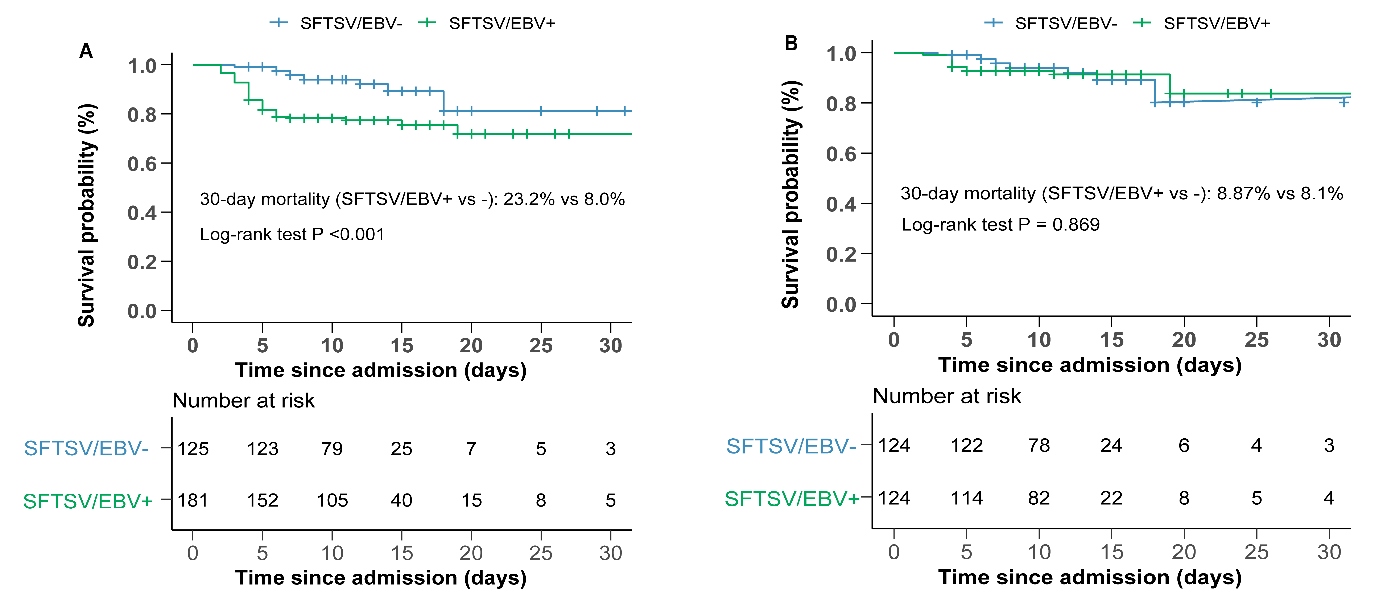


**Supplementary Figure 4. Comparison of immune cell counts (/μL) and cytokine levels between EBV-positive and EBV-negative SFTS patients. (ns, not significant, *P < 0.05, **P < 0.01, ***P < 0.001, ****P < 0.0001.)**

**
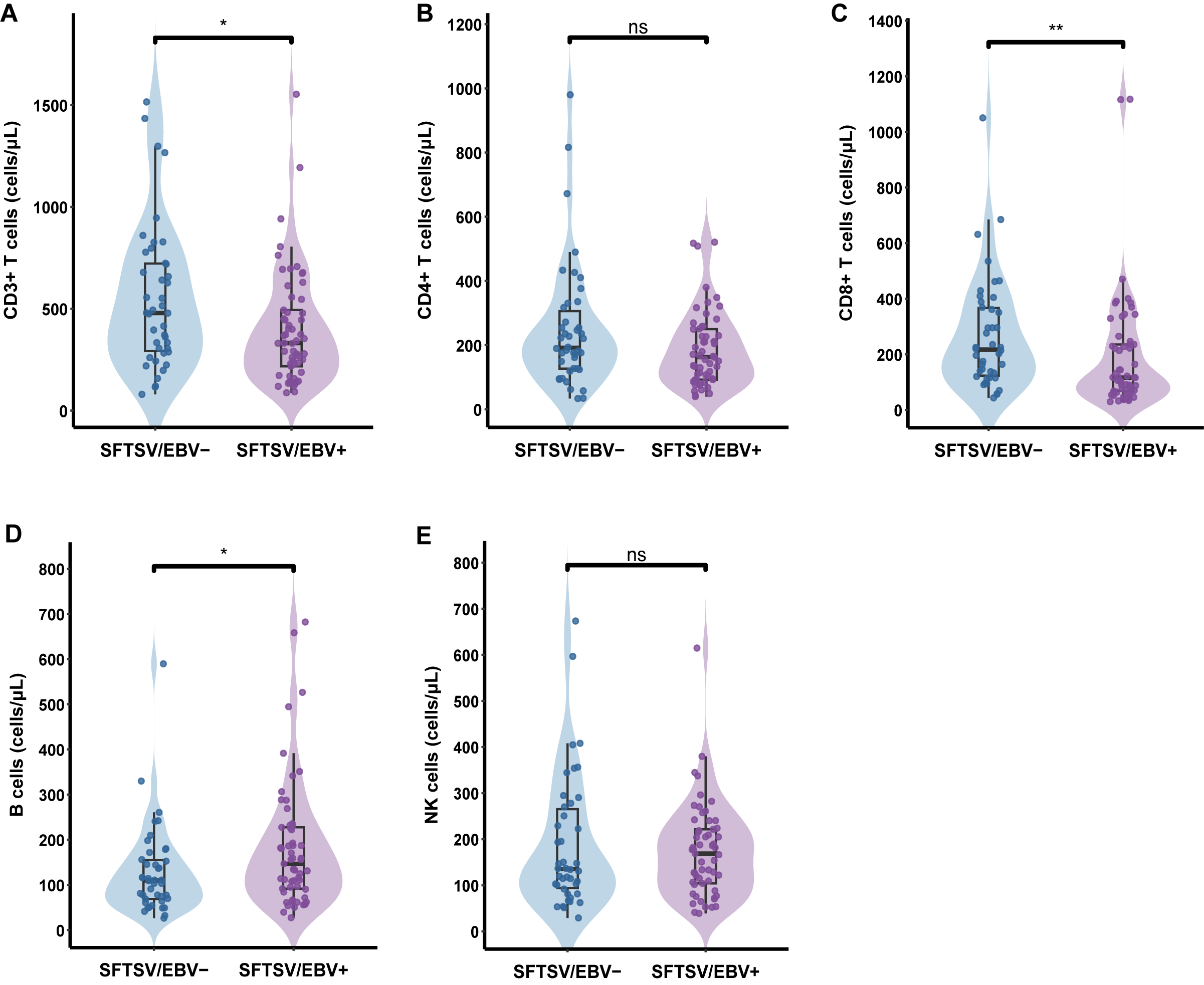
**

**Supplementary Figure 5. Comparison of cytokine levels between EBV-positive and EBV-negative SFTS patients. (ns, not significant, *P < 0.05, **P < 0.01, ***P < 0.001, ****P < 0.0001.)**

**
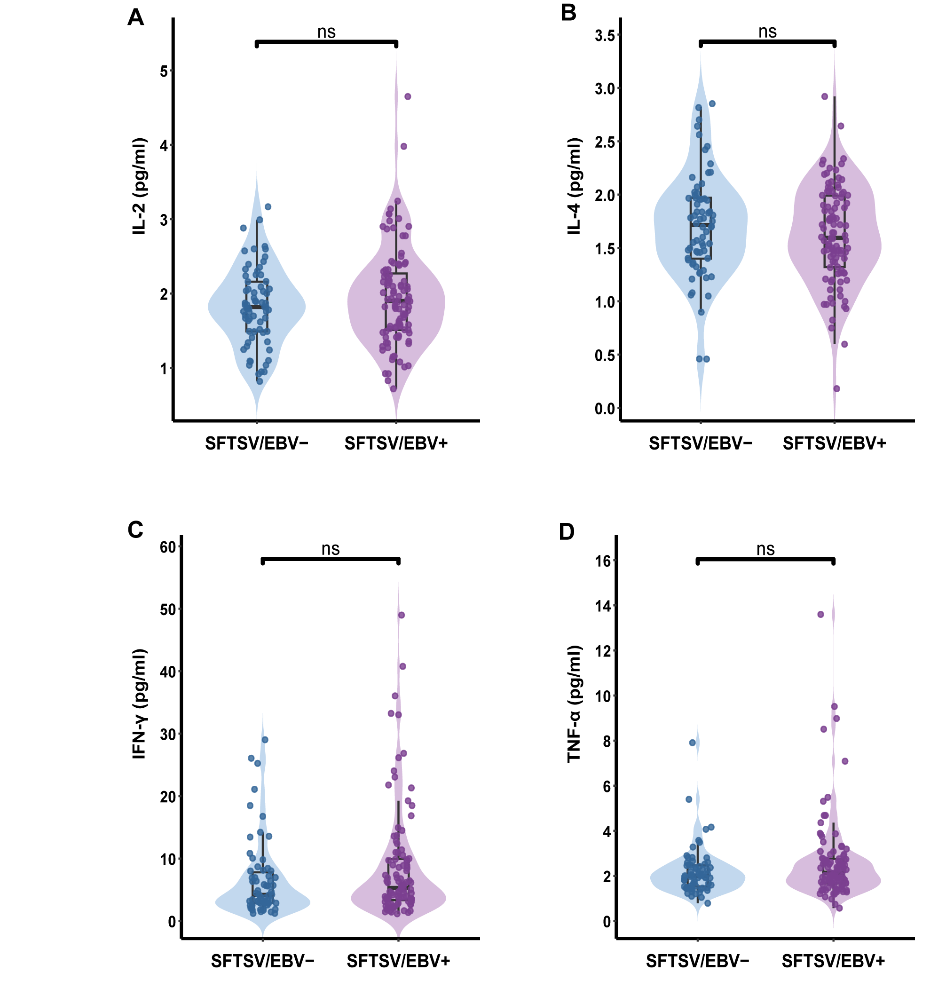
**
